# Molecular Approaches for Low-Cost Point-of-Care Pathogen Detection in Agriculture and Forestry

**DOI:** 10.3389/fpls.2020.570862

**Published:** 2020-10-28

**Authors:** Paolo Baldi, Nicola La Porta

**Affiliations:** ^1^IASMA Research and Innovation Centre, Fondazione Edmund Mach, Trento, Italy; ^2^The EFI Project Centre on Mountain Forests (MOUNTFOR), San Michele a/Adige, Trento, Italy

**Keywords:** biosensor portable devices, in field detection, qPCR, LAMP, RPA, RCA, SDA, MinION nanopore sequencing

## Abstract

Early detection of plant diseases is a crucial factor to prevent or limit the spread of a rising infection that could cause significant economic loss. Detection test on plant diseases in the laboratory can be laborious, time consuming, expensive, and normally requires specific technical expertise. Moreover, in the developing countries, it is often difficult to find laboratories equipped for this kind of analysis. Therefore, in the past years, a high effort has been made for the development of fast, specific, sensitive, and cost-effective tests that can be successfully used in plant pathology directly in the field by low-specialized personnel using minimal equipment. Nucleic acid-based methods have proven to be a good choice for the development of detection tools in several fields, such as human/animal health, food safety, and water analysis, and their application in plant pathogen detection is becoming more and more common. In the present review, the more recent nucleic acid-based protocols for point-of-care (POC) plant pathogen detection and identification are described and analyzed. All these methods have a high potential for early detection of destructive diseases in agriculture and forestry, they should help make molecular detection for plant pathogens accessible to anyone, anywhere, and at any time. We do not suggest that on-site methods should replace lab testing completely, which remains crucial for more complex researches, such as identification and classification of new pathogens or the study of plant defense mechanisms. Instead, POC analysis can provide a useful, fast, and efficient preliminary on-site screening that is crucial in the struggle against plant pathogens.

## Introduction

Plant pathogens represent one of the major threats for agriculture worldwide (Narayanasamy, 2011; Aslam et al., 2017; Pallas et al., 2018). In the last centuries, in parallel with the continuing growth of the human population, the percentage of the land surface covered by crops has increased constantly, with ca. 50% of the current habitable world land dedicated to agriculture^1^. In 2016, it was estimated that approximately 540 million hectares were planted worldwide to only three major crop species: maize, rice, and wheat (McDonald and Stukenbrock, 2016). Plant pathogens can cause substantial reduction of crop productivity. Considering five of the major crops (maize, rice, wheat, potatoes, and soybean), it was shown that the losses, associated with pathogens and pests at a global level, ranged between 17 and 30% annually (Ramakrishnan et al., 2019; Savary et al., 2019). In agriculture and forestry, most of the damage is due to the accidental introduction of invasive alien pathogen species into new areas, as a consequence of global trade and transport (Ghelardini et al., 2017). Moreover, the extension of the distribution range of the pathogens due to human-mediated activities facilitates hybridization and horizontal gene transfer, leading to the emergence of new pathogens (Gluck-Thaler and Slot, 2015). Also, the introduction of arthropod vectors into new areas can bring the establishment of novel association with introduced or native pathogen species (Wingfield et al., 2016). Well known examples of introduction of invasive pathogens exist both in agriculture and forestry. The wheat blast pathogen *Pyricularia graminis-tritici* appeared recently in Asia for the first time, maybe coming from South America, and devastated more than 15,000 ha of crops in Bangladesh (Callaway, 2016). In Italy, since 2013, *Xylella fastidiosa* is causing a severe loss of olive trees in the southern part of the country. Genetic analysis of the Italian strains showed similarity to isolates from Central America (Marcelletti and Scortichini, 2016; Giampetruzzi et al., 2017). *Cronartium ribicola*, the causal agent of white pine blister rust migrated from Europe to North America along with plant material, while the subspecies *americana* of the Dutch elm disease fungus *Ophiostoma novo-ulmi* arrived in Europe from North America with rock elm logs (Ghelardini et al., 2017).

Another factor that influences plant–pathogen interaction is climate change. Increased temperature, climate extremes, as well as differences in quantity and pattern of annual precipitation, can support the spread of plant diseases in agriculture (Schmidhuber and Tubiello, 2007) and forests (La Porta et al., 2008). Finally, the great majority of the cultivated soil is planted to monocultures or even to only one genotype (fruit trees, grape, and potato), thus creating a homogeneous genetic environment that can easily select host-specialized crop pathogens.

In order to control, and when possible to prevent, plant diseases and the spread of plant pathogens into new areas, it is mandatory to develop fast, efficient, and inexpensive methods for early detection of pathogens. Traditional methods for fungi and bacteria identification rely on symptom observation and culture-based methods (Alahi and Mukhopadhyay, 2017). This implies the necessity of growing the pathogen on specific media in controlled conditions, a process that usually takes days or even weeks. Moreover, the morphological identification of the pathogen by microscopy requires specifically trained personnel.

Other widely used phyto-diagnostic methods are based on immunological techniques such as enzyme-linked immunosorbent assay (ELISA) (Cho and Irudayaraj, 2013), immunofluorescent staining (Khater et al., 2017), immunoblot (Novakova et al., 2006), and lateral flow immunoassays (LFIA) (Burnham-Marusich et al., 2018; Cassedy et al., 2020). Nonetheless, the production of monoclonal antibodies may be expensive, and in some cases, problems of low specificity have been reported (Gorris et al., 1994; Martinelli et al., 2015). Since the nineties, more laboratories started to adopt DNA-based methods for pathogen detection and identification, using polymerase chain reaction (PCR) and its variants, such as quantitative PCR (qPCR), nested PCR, multiplex PCR, and digital PCR (ddPCR) (Martinelli et al., 2015). Such methods are usually very specific, relatively fast, and cheap but still present some disadvantages, as in most cases, specific equipment is required as well as trained personnel. Since the first few years of this century, new technologies are emerging based on isothermal amplification of DNA, such as the loop-mediated isothermal amplification (LAMP), recombinase polymerase amplification (RPA), helicase-dependent amplification (HDA), and others (Lau and Botella, 2017), capable of overcoming some of the drawbacks of PCR-based methods (Zhong and Zhao, 2018). All these approaches, even if based on different principles, share the main characteristic of amplifying target DNA at constant temperature, therefore eliminating the need of a thermal cycler.

Due to the rapid evolution of methods for pathogen detection, in many fields such as human health and food safety, it is dawning the possibility to develop new tools that can be taken directly to the desired analysis site, also called point-of-care (POC) (Chen et al., 2019; Vidic et al., 2019). Ideally, such tools should consist of robust, portable, user-friendly, and inexpensive equipment capable of performing all the steps required for a complete analysis in a short time and with a minimal number of steps, in order to be used also by low-trained personnel. All these requirements have been summarized for the first time by the World Health Organization (WHO) in the ASSURED (Affordable, Sensitive, Specific, User-friendly, Robust and rapid, Equipment-free, Deliverable) guidelines for POC testing (Peeling et al., 2006). The potential advantages of POC analysis are numerous (Table 1), going from the speed of the analysis to the lower risk of sample contamination and pathogen dispersion. As a matter of fact, the possibility to detect a pathogen (Radhakrishnan et al., 2019) directly in the field would be of great interest in agriculture and forestry. It would allow extensive and fast screening of plants in order to prevent disease spreading. Moreover, imported plants and products could be analyzed directly on-site before they can even enter a country. Two important applications of POC analysis are the study of emergent non-native introduced pathogens (Tomlinson et al., 2010; Buhlmann et al., 2013; Blaser et al., 2018; Aglietti et al., 2019; Rani et al., 2019) and chronic disease monitoring (De Boer and Lopez, 2012; Zheng et al., 2013).

**TABLE 1:** Main advantages of point-of-care (POC) analysis when compared to standard laboratory testing.

Low price
Faster diagnosis and crop decision-making
Low number of steps for the analysis
No storage needed
Low chance of sample deterioration or contamination
Implementable for Citizen Science
No laboratory facilities needed
Low chance of spreading diseases outside contained areas
No samples lost in transit
Very efficient for the analysis of imported plant material
Allows early detection and surveillance activity in epidemiological studies
Low-trained personnel needed
Easy remodeling of the sampling design by results in real time
Allows easy planning of pesticide treatments in the field

**FIGURE 1:**
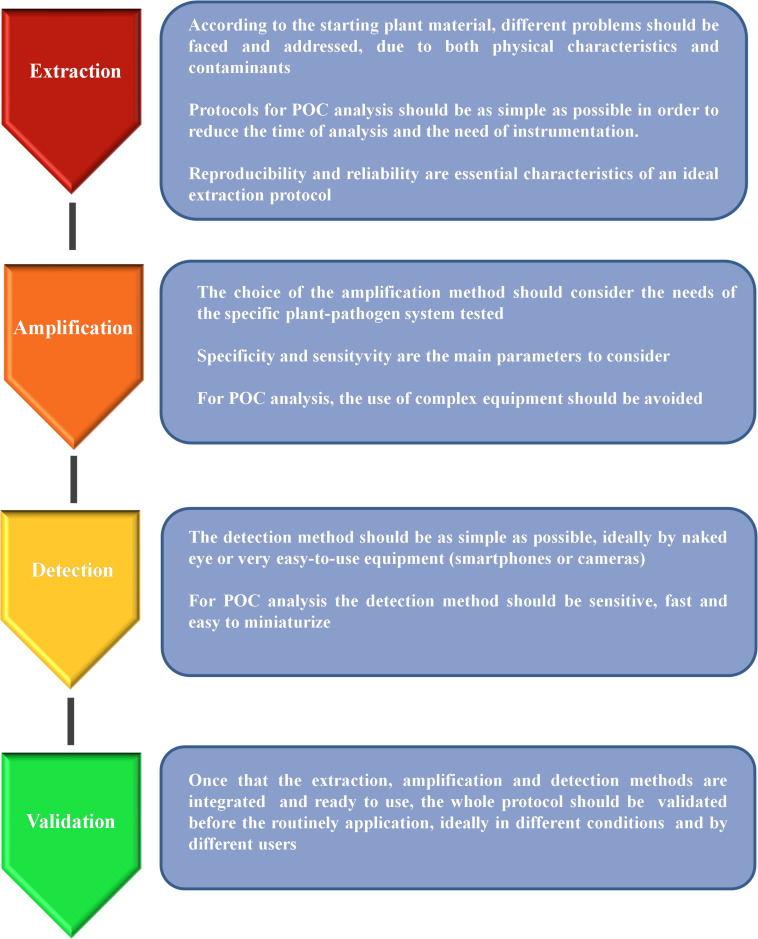
Schematic representation of the main steps needed for the development of a reliable protocol suitable for point-of-care (POC) analysis.

The DNA-based analysis methods are proving to be good candidates for the development of POC analysis protocols, especially since the advent of the new isothermal technologies. Nevertheless, providing a portable tool capable of performing a complete test in the field is not an easy task as there are at least three main steps that should be performed: (a) sample preparation/DNA extraction; (b) DNA amplification; (c) signal detection (Figure 1). In the following sections, we analyze the progress made in the recent years in order to exploit DNA-based technologies for the development of portable tools suitable for plant pathogen detection, considering all the main steps of the analysis and with a particular focus on their application in agriculture and forestry.

## Nucleic Acid Extraction

Despite the significant progress made to adapt the DNA amplification technologies to the needs of POC analysis of plant pathogens (Lau and Botella, 2017; Donoso and Valenzuela, 2018; Dairawan and Shetty, 2020), one of the main obstacles is plant sample preparation and nucleic acid extraction. As a matter of fact, a high-quality DNA or RNA is always necessary in order to obtain reliable and reproducible results, irrespective of the method used for nucleic acid amplification. Even if several methods have been developed for the fast extraction of nucleic acids from bacteria, animal cells, and food (Cheng and Jiang, 2006; Kim et al., 2009; Cawthorn et al., 2011; Abdalhai, 2016), such technologies are often difficult to apply to plant tissues. If we consider a leaf, the most common plant tissue used for nucleic acid extraction, cells are surrounded by several protective structures, such as cell wall and waxy cuticle layers. All these structures must be degraded in order to extract the genetic material inside, therefore adding a level of complexity to the protocol. Moreover, depending on the different plant species and tissues, a lot of plant contaminant products can be present, such as proteins, polysaccharides, polyphenolics, and a great number of secondary metabolites (Varma et al., 2007). All these contaminants should be removed from the genetic material in order to obtain a high-quality template suitable for amplification. Several strategies can be applied, such as the removal of cellular proteins by organic solvents (typically phenol and chloroform), but all these steps are quite difficult to include in a completely portable device.

Many different solutions have been proposed (Table 2), the simpler of which is the use of plant crude extract (ground plant material with an extraction buffer) directly for amplification. Munawar et al. (2019) used strawberry crude crown tissue macerated with a standard ELISA grinding buffer coupled with RPA for the detection of *Phytophthora cactorum* infecting strawberry. Successful detection of the pathogen was achieved, after testing several concentrations of the grinding buffer, in order to optimize amplification also in plants showing early or no symptoms. Several works showed comparison in amplification efficiency between purified templates and crude extracts. For the detection of *Xanthomonas fragariae*, the causal agent of angular leaf spot of strawberry, a test was carried out using two types of templates: pure bacterial DNA and crude extract (Getaz et al., 2017). LAMP DNA amplification was successful with crude tissue extract as well as with the pure bacterial DNA control, after optimization of the protocol. The optimized protocol with phenol inhibitors was also successfully tested in naturally infected strawberry leaves, even those with weak symptoms, therefore suggesting that this method could be a suitable POC analysis. In another work, different plant sample preparation methods were compared, using sap obtained after grinding apricot (*Prunus armeniaca*) plant tissues as a starting material for the detection of *Candidatus phytoplasma prunorum* (Minguzzi et al., 2016). Similarly, several grinding buffers were compared for *Fragaria ananassa* crude sample preparation for the rapid detection of *Phytophthora* spp. (Miles et al., 2015). All the above reported results seem to indicate that the use of plant crude extract for the direct detection of pathogens with nucleic acid amplification technologies can be effective and therefore used for in-field application. The advantage of crude extracts is essentially due to the very simple and fast extraction protocols, while the main drawback is the possible lack of sensitivity. Therefore, a preliminary testing of the method is always necessary, together with an appropriate optimization according to the plant species, tissue used as starting material, and specific primers designed for the amplification step.

**TABLE 2:** List of nucleic acid extraction methods suitable for POC analysis.

Extraction method	Advantages	Disadvantages	Applied for plant pathogens	References
Crude extract	Fast, inexpensive, no need of trained personnel	Presence of contaminants, lack of sensitivity and reproducibility	Yes	Miles et al. (2015); Minguzzi et al. (2016); Getaz et al. (2017); Munawar et al. (2019)
Magnetic beads	Reliable, high-quality genetic material, no need of centrifugation	Expensive, not completely user friendly	Yes	Chang et al. (2013); Schwenkbier et al. (2015b); Lin et al. (2015); DeShields et al. (2018); Wei et al. (2018); Lei et al. (2019)
Piercing methods	Fast, inexpensive, no need of trained personnel	Lack of sensitivity, necessity of optimization	Yes	Fukuta et al. (2005); Suzuki et al. (2016); Paul et al. (2019, 2020); Wilisiani et al. (2019)
Cellulose filter paper	Fast, inexpensive, no need of trained personnel	Possible absorption of inhibitors, necessity of optimization	Yes	Lu et al. (2016); Zou et al. (2017)

Sensitivity and specificity of pathogen detection protocols can be improved by purifying target DNA. There are a lot of commercial kits available for this purpose, but most of them require to be used in a laboratory with dedicated equipment. Nonetheless, several commercial kits have been tested, and some of them showed characteristics that could be suitable for POC analysis, especially those based on the use of magnetic beads for nucleic acid purification (DeShields et al., 2018). Several works can be found in literature exploiting this extraction principle on various plant species and pathogens, such as detection of the fungus *Leptosphaeria maculans*, causing Phoma stem canker (black leg) of *Brassica napus* (Lei et al., 2019). Other examples include detection of soil-borne pathogens of potatoes (DeShields et al., 2018); *Alternaria panax* Whetz infecting ginseng (Wei et al., 2018); *Puccinia striiformis* f.sp. *tritici* (wheat yellow rust) infecting wheat (Radhakrishnan et al., 2019); *Phytophthora kernoviae* infecting *Rhododendron ponticum* (Schwenkbier et al., 2015b) and several other species of *Phytophthora* (Julich et al., 2011; Schwenkbier et al., 2014). It must be noted that in some of the above reported cases, nucleic acid extraction was performed from cultivated pathogens and not directly from infected plants, therefore suggesting that optimization of the protocol could be necessary before its routine application in the field. A magnetic bead-based RNA extraction method was used to develop a microfluidic chip for the detection of viruses infecting *Phalaenopsis orchid* (Chang et al., 2013; Lin et al., 2015). The main advantage of the magnetic bead-based extraction method is reliability, as most of the more commonly used protocols exploit robust commercial kits already available on the market. Moreover, it is possible to extract genetic material without the need of centrifugation steps, but simply using a magnet that can be easily transported and used on site even when electricity supply is not available. One possible drawback is that most of the commercial kits are not 100% user friendly, and some training is still necessary to use them properly and avoid contamination. In order to overcome this problem, some authors tried to simplify the extraction protocol, still using commercial kits but introducing some modifications. The first report describing a complete protocol for plant pathogen detection that could be performed directly in the field dates back to 2005, and the DNA was extracted modifying a commercial kit in order to avoid the use of liquid nitrogen and laboratory equipment, such as centrifuge and vortex (Tomlinson et al., 2005). Similarly, other authors proposed a modification to standard protocols to simplify the extraction of genetic material suitable for amplification with various techniques (Li et al., 2011; Koo et al., 2013).

A very simple method proposed for DNA or RNA extraction is the so-called “toothpick method,” in which a sterile toothpick is used to pierce the sample tissue several times. The tip of the toothpick is then dipped directly in the amplification reaction mix, releasing target genetic material (Fukuta et al., 2005; Suzuki et al., 2016; Wilisiani et al., 2019). Despite the extreme simplicity, this method needs to be tested and optimized for each specific need; an example is provided in the next lines. A modern version of the “toothpick method” was recently proposed using disposable polymeric microneedle patches (MP) to pierce plant leaves. These patches are made of polyvinyl alcohol (PVA), a water-absorbing polymer that showed a good combination of mechanical strength, chemical resistance, and biocompatibility (Paul et al., 2019, 2020). The use of MP to detect *Phytophthora infestans* from tomato leaves showed a slightly lower sensitivity in comparison to the standard CTAB extraction method, especially during the very early stages of infection. On the other hand, the extraction time was reduced to 1 min, instead of 3–4 h of the CTAB method, suggesting that MP could be easily used for in-field applications.

In the last few years, the development of new nucleic acid extraction methods for POC analysis has increased, exploring different approaches. Zou et al. (2017) tested a variety of materials for their ability to capture nucleic acids and found that Whatman No. 1 cellulose-based filter paper can efficiently entrap and retain DNA without the need of any chemical treatment. Filter paper-based extraction methods were positively tested on different plant species, such as wheat, rice, tomato, soybean, tobacco, mandarin, and lemon, showing good sensitivity and reproducibility. Similarly, Flinders Technology Associates (FTA^®^ Card, Whatman^TM^, catalog number: WB120205, Merck KGaA, Darmstadt, Germany) cards were used for extraction of DNA or RNA from different starting materials, including plants, to provide a versatile POC pathogen analysis method (Lu et al., 2016). Paper-based microfluidics device has emerged as a multiplexable POC platform, which might be useful in resource-limited settings (Yetisen et al., 2013). This device was applied in health care, veterinary medicine, food safety, and environmental and crop monitoring.

A rapid technique for the extraction of viral RNA from rose plants was also proposed, based on the direct virus absorption onto PCR tubes at 4°C (Babu et al., 2017). This extraction method provided viral RNA suitable for molecular analysis in as few as 5 min. This method is very fast and simple, but more studies are still necessary to understand if it could be applicable routinely to different pathogens and plant species.

Another option for the rapid extraction of nucleic acids from plant tissue is the use of lateral-flow devices (LFDs). A method was developed allowing the extraction of amplifiable DNA from plant tissue using LFD in as few as 5 min without the use of any equipment (Danks and Barker, 2000). A simplified version of this method was described and applied to the detection of *Phytophthora ramorum* and *Phytophthora kernoviae* (Tomlinson et al., 2010).

## PCR-Based Nucleic Acid Amplification

Once that the DNA or RNA is extracted, the next step is the amplification of the genetic material using specific primers designed to recognize target sequences of the pathogen. Several approaches can be used and are listed in Table 3. The most popular methods for laboratory nucleic acid amplification are still PCR-based ones, including many variants such as quantitative real-time PCR (qPCR), nested PCR, and digital PCR. Nevertheless, when rapid and simple POC amplification is required, PCR may present some drawbacks, as it usually requires a thermal cycler, a device that is not easy to miniaturize and integrate in a portable tool. Still, some attempts in this direction have been made for POC application in agriculture. Julich et al. (2011) developed a lab-on-a-chip device for rapid nucleic acid-based detection of *Phytophthora* species. Temperature management in the PCR and hybridization zones relies on two independent Peltier elements. The system is completed by a compact portable pump and power supply for use on site. A similar approach was proposed by Schwenkbier et al. (2014) who described a lab-on-a-chip device for on-site detection of *Phytophthora* species, exploiting linear-after-the-exponential PCR (LATE-PCR) as amplification method.

**TABLE 3:** List of the main polymerase chain reaction (PCR)-based amplification methods applied for POC plant pathogen detection.

Method	Target	Advantages	Disadvantages	References
Conventional PCR	DNA/RNA	Reliable, relatively cheap	Time consuming, requires a thermocycler	Julich et al. (2011); Schwenkbier et al. (2014)
Nested-PCR	DNA/RNA	Very specific	Expensive, time consuming, requires a thermocycler	Costa et al. (2012); Wei et al. (2018)
Real-time PCR	DNA/RNA	Allows absolute quantification of DNA; no need of post-amplification detection	Time consuming, requires highly purified genetic material, requires a thermocycler	Tomlinson et al. (2005); Arif et al. (2013); Koo et al. (2013); Baidoo et al. (2017); Bini et al. (2018); DeShields et al. (2018); Fotiou et al. (2019)
Digital PCR (ddPCR)	DNA/RNA	High sensitivity, resilient to contaminants, allows absolute quantification of DNA	Expensive, requires specific equipment	Selvaraj et al. (2018); Voegel and Nelson (2018); Zhong et al. (2018); Liu et al. (2019)

Also, qPCR was often proposed as suitable for POC analysis. One of the first reports dates back to 2002 (Schaad et al., 2002) when a protocol for 1-h on-site diagnosis of *Xylella fastidiosa* was described using a portable Smart Cycler. Other examples of qPCR-based methods for POC pathogen detection include a system for the detection of *Phymatotrichopsis omnivora*, in alfalfa and other dicot plants (Arif et al., 2013) and a protocol for the detection of *Phytophthora ramorum*, an invasive plant pathogen listed under quarantine in many countries and capable of infecting over 180 forest trees species (Tomlinson et al., 2005). Papers describing fast protocols for plant pathogen detection via qPCR that could be potentially used for in-field applications have been published for several species such as *Plum pox virus* (Fotiou et al., 2019), *Spongospora subterranea* (DeShields et al., 2018), *Austropuccinia psidii* (Bini et al., 2018), and *Pratylenchus penetrans* (Baidoo et al., 2017). In some cases, real-time-based protocols were proposed for multiplex detection of plant pathogens (Papayiannis, 2014; Khan et al., 2015; Yang et al., 2015; Osman et al., 2017; Nikitin et al., 2018). A recent study proposed an *In Situ* Processing and Efficient Environmental Detection (iSPEED) kit to detect pests and pathogens of forest trees using POC qPCR (Capron et al., 2020). Targeted diseases included the poplar canker pathogen *Sphaerulina musiva*, the white pine blister rust *Cronartium ribicola*, the comandra blister rust of pines *C. comandrae*, the Port Orford cedar pathogen *Phytophthora lateralis*, and the agent responsible for sudden oak and sudden larch death *P. ramorum*. The iSPEED kit could be carried in a small backpack and showed results comparable to those obtained with standard laboratory equipment. The advantages of the iSPEED kit are the existence of a great amount of published and validated real-time PCR assays, multiple enzymes and chemistry for qPCR, as well as a growing range of portable instruments, cost effectiveness, and flexibility of design. Another variant of conventional PCR that can be used to increase specificity and sensitivity is nested-PCR. The use of two pairs of specific primers usually make this approach more time consuming and expensive than conventional PCR. Nevertheless, some researchers have developed a single-tube nested-PCR approach that potentially eliminates the drawbacks of this technique, such as contamination of the sample (Costa et al., 2012; Wei et al., 2018).

Finally, droplet digital PCR (ddPCR) is a new technology allowing absolute quantification of target DNA, which is partitioned into approximately 20,000 droplets. The amplification reaction is carried out independently within each droplet, and a reader can estimate the amount of amplified DNA in each droplet by fluorescence measurement (Hindson et al., 2011). ddPCR offers many advantages when compared to conventional PCR and even qPCR, as multiple target genes can be detected in a single reaction using different fluorescence signals. Moreover, no standard curve is required for quantification, and the sensitivity of ddPCR was shown to be higher than quantitative PCR when measuring low copy-number genes (Hayden et al., 2013). Another advantage is the high resilience of ddPCR to contaminants, that can present a major problem when plant samples are analyzed (Racki et al., 2014; Blaya et al., 2016). Although ddPCR has not been used for POC analysis in agriculture yet, some approaches using this method have been described (Selvaraj et al., 2018; Zhong et al., 2018; Liu et al., 2019), and in one case, the ddPCR protocol was proposed to be applied for use in national clean plant programs to prevent the import of infected nursery stock (Voegel and Nelson, 2018).

## Isothermal Nucleic Acid Amplification

In recent years, new technologies for isothermal amplification of nucleic acids have been developed, and their use is rapidly spreading (Table 4). The first and most commonly used is loop-mediated isothermal amplification (LAMP) (Notomi et al., 2000). The main advantages of LAMP when compared to PCR-based approaches are the possibility to perform the amplification reaction at constant temperature, the short reaction time, high amplification efficiency, and sensitivity, combined to a relatively low cost, as LAMP requires very simple equipment to be performed (Zhao et al., 2015; Lai et al., 2018). Several portable devices for POC detection of plant pathogen have been described exploiting LAMP technology. A rapid and simple method to identify three different species of begomovirus infecting *Cucurbitaceae* and *Solanaceae* plants was recently published, using a commercial portable device that is battery powered, user friendly, handheld, and waterproof (Wilisiani et al., 2019). Another example of portable device suitable for agriculture and forestry application is a micropipette tip-based nucleic acid test (MTNT) capable of detecting both DNA and RNA from different plant crude extracts (Lu et al., 2016). An increasing number of fast LAMP protocols for POC detection of plant pathogens are being developed for several species including forest trees (Tomlinson et al., 2010; Yaseen et al., 2015; Aglietti et al., 2019) as well as herbaceous plants (Chang et al., 2013; Lin et al., 2015; Ammour et al., 2017). All these protocols combined fast nucleic acid extraction methods and signal detection to LAMP in order to perform all the required steps directly in the field. It must be noted that, although LAMP is actually a very promising technique for POC analysis (Notomi et al., 2015), it still presents some limitations, such as the design of the specific primers, that sometimes can be difficult. Several authors have developed fast LAMP protocols that, even if not yet incorporated in a specific portable device, were evaluated to be simpler, faster, cheaper, and with higher sensitivity when compared with traditional methods (Kikuchi et al., 2009; Tomlinson et al., 2010; Villari et al., 2013). By the optimization of RT-LAMP assay, a protocol was developed for the detection of *B. xylophilus*, a pine nematode. Such protocol can distinguish between living and dead nematodes, by detecting the presence of mRNA encoding an expansin gene as a viability marker (Leal et al., 2014). Other examples of optimized LAMP-based protocols include a tool for the early detection of *Heterobasidion irregulare* (Sillo et al., 2018); a fast, easy, and in-field deployable method for detection of *Pepino mosaic virus* and *Potato spindle tuber viroid* (Mehle et al., 2017); a LAMP protocol starting from crude plant materials for the detection of *Xanthomonas fragariae* (Getaz et al., 2017); a protocol for the detection of three different phytoplasmas infecting fruit trees (De Jonghe et al., 2017); and an assay for the rapid detection of *Pectobacterium atrosepticum* (Li et al., 2011). Similar to PCR-based approaches, multiplexing of the samples is possible with LAMP (Denschlag et al., 2014; Yasuhara-Bell et al., 2018; Feng et al., 2019).

**TABLE 4:** List of the main isothermal amplification methods applied for POC plant pathogen detection.

Method	Target	Advantages	Disadvantages	References
Loop-mediated isothermal amplification (LAMP)	DNA/RNA	Fast, isothermal, high sensitivity, relatively cheap	Primer design can be difficult	Tomlinson et al. (2010); Chang et al. (2013); Lin et al. (2015); Yaseen et al. (2015); Lu et al. (2016); Ammour et al. (2017); Aglietti et al. (2019); Wilisiani et al. (2019)
Recombinase polymerase amplification (RPA)	DNA/RNA	Fast, isothermal, does not require an initial denaturation step	Long primers needed, specificity and sensitivity may vary	Zhang et al. (2014); Hammond and Zhang (2016); Ahmed et al. (2018); Gaige et al. (2018); Burkhardt et al. (2019); Strayer-Scherer et al. (2019)
Rolling circle amplification (RCA)	DNA/RNA	Isothermal, high sensitivity and specificity	Expensive, the detection may be tricky	Davari et al. (2012); Rezk et al. (2019)
Displacement amplification (SDA)	DNA/RNA	Fast, isothermal	Inefficient for amplification of long transcript	Song et al. (2018); Venzac et al. (2018)
Helicase-dependent amplification (HDA)	DNA	Fast, isothermal, does not require an initial denaturation step	High optimization needed	Schwenkbier et al. (2015a,b); Wu et al. (2016)
Nucleic acid sequence-based amplification (NASBA)	RNA	Fast, isothermal	Expensive	Olmos et al. (2007); Smith et al. (2007); Scuderi et al. (2010); Tsaloglou et al. (2011); Dobnik et al. (2014)

Another recently developed isothermal amplification technique is the so-called recombinase polymerase amplification (RPA) (Piepenburg et al., 2006). RPA is becoming a common choice when POC analysis is required for application in agriculture (Ahmed et al., 2018; Burkhardt et al., 2019; Strayer-Scherer et al., 2019) and forestry (Cha et al., 2020), as it presents several advantages when compared to PCR and even to LAMP. In fact, RPA does not require an initial heating step for DNA denaturation, as it exploits enzymatic activity to separate the double strand. Moreover, the reaction temperature is quite low (37 to 42°C), and the reaction time is usually very short (Kappagantu et al., 2017; Munawar et al., 2019). When RPA was compared to RT-LAMP as a detection method using a small (150 mm × 200 mm × 35 mm) and light (400 g) battery-mounted portable optical isothermal device (Cha et al., 2020), the detection limit of RPA was shown to be 10 times lower than RT-LAMP. All these characteristics make RPA a very easy-to-use approach, especially in developing countries when fast analysis in low resource environments is needed (Wambua et al., 2017; Ghosh et al., 2018; Silva et al., 2018). The possibility to multiplex the reaction can further increase the speed and reduce the costs of RPA (Lau et al., 2016; Lei et al., 2019). Several RPA-based portable devices are already available for POC detection of human pathogens (Ereku et al., 2018; Tsaloglou et al., 2018), and assays for plant pathogen detection are rapidly developing (Zhang et al., 2014; Hammond and Zhang, 2016; Gaige et al., 2018). An integrated cheap prototype platform termed POCKET (Point-Of-Care Kit for the Entire Test) was recently developed demonstrating that combining 3D printing, microfluidics, RPA, and a smartphone, it is possible to create an ultraportable, inexpensive, and versatile device for analyzing multiple types of DNA from clinics to environment to food to agriculture in a sample-to-answer manner (Xu et al., 2020). The POCKET device is less than 100 g and smaller than 25 cm in length.

However, RPA still presents some limitations: it allows amplification of only small DNA fragments (<500 bp) with long primers (30–35 nt), resulting sometimes in non-specific amplification (Lau and Botella, 2017) and a highly variable sensitivity (Miles et al., 2015; Babu et al., 2017; Rojas et al., 2017).

Several other techniques for the isothermal amplification of nucleic acid exist such as rolling circle amplification (RCA), strand displacement amplification (SDA), helicase-dependent amplification (HDA), and nucleic acid sequence-based amplification (NASBA). All such techniques have characteristics that can be suitable for POC analysis in agriculture, and even if at present only a few examples of their use in field conditions exist, they are evolving rapidly. RCA was recently proposed for rapid diagnostic use in human health (Cao et al., 2019; Soares et al., 2019; Zhang et al., 2019), and some portable devices have been developed (Yao et al., 2018). Several examples of the use of RCA for plant pathogen detection exist (Davari et al., 2012; Rezk et al., 2019), but no portable devices have been described yet for application in agriculture. For SDA, some portable devices have been recently developed for human health care (Song et al., 2018; Venzac et al., 2018), so it is more than conceivable that in the next few years, this technique will be applied also for POC analysis in plant pathogen detection.

Another promising isothermal technique for amplification of genetic material is HDA, which was used in agriculture for the detection of tomato spotted wilt virus (Wu et al., 2016) and *Phytophthora* species (Schwenkbier et al., 2015a,b). Finally, NASBA has already found application in plant pathogen detection (Olmos et al., 2007; Scuderi et al., 2010; Dobnik et al., 2014), but it has not yet become a standard method for this kind of analysis, especially when POC pathogen detection is required, even though several portable devices have been developed and described (Smith et al., 2007; Tsaloglou et al., 2011). Some of the possible reasons could be that NASBA is not yet a widely known technique and the relatively high cost of the reactions (Honsvall and Robertson, 2017).

## Detection Methods

In order to detect the amplification products efficiently, various methods can be employed (Table 5). In particular, for POC tests, the detection systems should require as few equipment as possible to facilitate the analysis in the field. Most of the devices proposed for POC analysis use optical or visual tools (Zhang et al., 2019). Different principles can be exploited for the optical detection of amplicons, such as fluorescence, chemiluminescence, and colorimetric visualization among others. The signal can be read by the naked eye or by the use of standard optical instruments including CCD cameras, smartphones, and light detectors.

**TABLE 5:** List of nucleic acid detection methods suitable for POC analysis.

Detection method	Advantages	Disadvantages	Applied for plant pathogens	References
Fluorescence-based	Reliable, high sensitivity, easy to integrate into microfluidic devices	Expensive, cannot be detected by naked eye	Yes	Koo et al. (2013); DeShields et al. (2018); Larrea-Sarmiento et al. (2018); Fotiou et al. (2019); Lei et al. (2019); Tahzima et al. (2019); Xia et al. (2019); Zhang et al. (2019)
Surface-enhanced Raman scattering (SERS)	High sensitivity, easy to multiplex	Expensive, needs optimization, cannot be detected by naked eye	Yes	Lau et al. (2016)
Colorimetric	Low cost, low equipment requirement, easy to integrate into microfluidic devices	Lack of sensitivity and selectivity	Yes	Chang et al. (2013); Rigano et al. (2014); Kolm et al. (2015); Lin et al. (2015); Wei et al. (2018)
Bioluminescence assay in real-time (BART)	Real-time reading, easy data interpretation, good tolerance to inhibitors	Expensive, cannot be detected by naked eye, needs optimization	No	Kiddle et al. (2012) (GMO)
Electrochemical	High sensitivity, simple instrumentation requirement, easy to miniaturize	Sensitivity to electrochemically active samples	Yes	Julich et al. (2011); Schwenkbier et al. (2014, 2015b); Liu et al. (2020)
Magnetic	Fast, high sensitivity, easy to integrate into portable devices	Not yet developed for plant analysis	No	Lee et al. (2014); Orlov et al. (2016); Khater et al. (2017)
MinION	High-throughput results, easy detection of multiple pathogens	Not completely user friendly	Yes	Liau et al. (2019); Shaffer (2019)

The fluorescence-based detection methods have been widely employed for many years for the visualization of qPCR amplification products, using SYBR green or TaqMan probes to generate the signal (Holland et al., 1991). Simplicity and high sensitivity are the main advantages of fluorescence-based detection methods, together with the possibility of coupling fluorescence detection tools to microfluidic devices (Neethirajan et al., 2011). Several examples of portable devices exploiting fluorescence detectors for POC plant pathogen analysis can be found. Such devices combine fluorescence detectors with several amplification methods, such as qPCR (Koo et al., 2013; DeShields et al., 2018; Fotiou et al., 2019), LAMP (Larrea-Sarmiento et al., 2018; Tahzima et al., 2019; Xia et al., 2019), RCA (Zhang et al., 2019), and RPA (Lei et al., 2019). A possible alternative to the classical fluorescence-based detection methods is the so-called surface-enhanced Raman scattering (SERS), a technique that exploits the signal enhancement given by metal nanoparticles surfaces upon laser excitation (Kneipp et al., 1997). SERS has the potential to guarantee higher performance than fluorescence-based approaches, especially for multiplex analysis, due to its narrow and distinct spectral peaks and the possibility to use different labeled Raman reporters (Laing et al., 2016). Indeed SERS has been proposed as a technique of choice for the realization of a POC tool for multiplex plant pathogen detection (Lau et al., 2016). SERS was used in combination with RPA for the early detection of several plant pathogens infecting *Arabidopsis thaliana* before any visible symptom or at early stages of infection (Lau et al., 2016).

The colorimetric methods represent an interesting alternative to the fluorescence-based detection. Colorimetric sensing can be exploited for a number of applications as it presents many advantages, such as low costs, low equipment requirements, possibility of multiplexing, and easy integration with microfluidic devices. Moreover, it is possible to detect the signal by the naked eye or using very common tools as smartphones and cameras (Ong and Poljak, 2020). The change in color, brightness, or intensity can be obtained using different methods that generally include a reaction triggered by the presence of the target amplification product. In agriculture, the application of colorimetric testing has been proposed for POC analysis in several species and using different methods. In some cases, amplification products are labeled using biotinylated primers and specifically detected by simple devices as lateral flow dipsticks (Tomlinson et al., 2010; Zhang et al., 2013; Rigano et al., 2014; Kolm et al., 2015; Wei et al., 2018). Another colorimetric method proposed for application in POC plant pathogen detection exploits the formation of a large amount of pyrophosphate ions as a byproduct of LAMP reactions (Chang et al., 2013; Lin et al., 2015). Several other methods have been exploited to perform POC colorimetric detection of nucleic acids for plant or human pathogen detection, as for example, the use of hydroxyl naphthol blue (HNB) (Harper et al., 2010), the activation of peroxidase-like deoxyribozyme (PDz) (Reed et al., 2019), pH-sensitive dyes (Hamidi and Perreault, 2019), and silver enhancement (Dharanivasan et al., 2019). Despite many advantages, the colorimetric detection methods still present some drawbacks, mainly the lack of sensitivity and specificity.

Another principle that can be applied for nucleic acid detection is bioluminescence. In particular, a recently described bioluminescence assay in real time (BART) can detect continuously the exponential increase in inorganic pyrophosphate (PP_*i*_) produced during an isothermal amplification reaction such as LAMP (Gandelman et al., 2010). The big advantage of BART is that it allows to simplify greatly data interpretation and hardware requirements. This characteristic make BART a good candidate for POC detection of amplified nucleic acids. A combination of LAMP and BART reactions was used for GMO detection, showing a high sensitivity level and good tolerance to inhibitors, proving to be suitable for field application (Kiddle et al., 2012).

A less developed, but still interesting, method for nucleic acid detection is based on electrochemistry (Hnaiein et al., 2008; Deiss et al., 2011). This approach showed high sensitivity and the possibility of miniaturizing all the components of the device very easily, making the electrochemical detection method a good candidate for the development of portable tools for POC analysis of plant pathogens. Another advantage of the electrochemical approach is the independence from background light or sample color, even though electrochemically active samples may influence the readout. Examples of portable devices for the electrochemical detection of amplified DNA from plant pathogens are already available (Julich et al., 2011; Schwenkbier et al., 2014, 2015a). The method exploits the enzymatic-triggered silver deposition on the electrode to read the signal both visually and electrochemically.

It is worth to mention magnetic sensing as a promising technique that could be potentially applied to POC analysis of amplified genetic material. This technique requires the labeling of the target sequence with magnetic nanoparticles and the detection of their stray field by the use of highly sensitive magnetic sensors (Lee et al., 2014; Orlov et al., 2016; Khater et al., 2017). The main advantages of magnetic sensing techniques are the high sensitivity, fast analysis time, and the possibility to be integrated onto handheld, portable on-chip systems and microfluidic devices (Devkota et al., 2015; Giouroudi and Kokkinis, 2017).

## POC Sequencing

When the etiological agent is not known, the pathogen identification must rely on the use of an untargeted screening method by extracting, preparing, and sequencing all of the genomic material in a particular sample at once (Yeh et al., 2019). Even though POC sequencing is not routinely applied for plant pathogens yet, in the last few years, some portable sequencers have been developed that allow to perform amplicon sequencing directly in the field. The application value of portable sequencers was illustrated for the first time during emergent outbreaks of the Ebola and Zika virus in West Africa (Quick et al., 2016, 2017). Genome sequencing of the virus was carried out *in situ* by the first portable MinION sequencer [Oxford Nanopore Technologies (ONT) Ltd., Didcot, United Kingdom]. MinION is a portable, real-time device for DNA and RNA sequencing. Each consumable flow cell can generate as much as 30 Gb of DNA sequence data or 7–12 million reads when analyzing RNA. Ultra-long reads (hundreds of kb) are possible. Data are streamed in real time so that analysis can be performed during the experiment, and workflows are fully versatile. The MinION weighs under 100 g and plugs into a PC or laptop using a high-speed USB 3.0 cable, or used alongside the MinIT device for real-time analyses. The MinION sequencer is commercially available on the market^2^ with a price that is lower than 1,000 USD (Solares et al., 2018). Using DNA barcodes, useful for species discovery and species identification, it was estimated that up to 1,000 barcodes can be generated in one flow cell and that the cost per barcode can be <2 USD (Srivathsan et al., 2018). Speed is an important characteristic of MinION, as it is possible to perform the entire workflow from sample preparation, through DNA extraction, sequencing, bioinformatics, and interpretation in 2.5 h (Loit et al., 2019). Another advantage of MinION is that it can be easily coupled with different techniques to perform both targeted and untargeted analysis. For targeted analysis, both PCR-based and isothermal amplification can be used (Yamagishi et al., 2017; Radhakrishnan et al., 2019). Whole-genome amplification can be obtained by multiple displacement amplification (MDA), a technique that uses the high-fidelity phi29 polymerase combined with random hexamer primers to amplify DNA in isothermal reaction (Spits et al., 2006). Due to its portability, affordability, and speed in data production, the MinION sequencer found many applications, and it was used on a mountain, in a jungle, in the arctic, and on the International Space Station (Castro-Wallace et al., 2017). Recently, it was successfully applied for sequencing and assembly of a human genome with ultralong reads (Jain et al., 2018). It was shown to be suitable for COVID-19 fast diagnosis, and this platform is going to be further extended for diagnosing other viruses and pathogens (Wang et al., 2020).

In plants, an open- source workflow for long-read sequences was established in *Eucalyptus*. It was able to reliably and repeatedly obtain >6.5 Gb of long−read sequencing data with a mean read length of 13 kb and an N50 of 26 kb (Schalamun et al., 2019). Other examples of MinION application in agriculture and forestry include detection of pathogenic fungi (Cui et al., 2019; Hu et al., 2019; Rajarammohan et al., 2019; Srivastava et al., 2019; Purushotham et al., 2020), bacteria (Badial et al., 2018; Krehenwinkel et al., 2019; Zervas et al., 2019; Fujiyoshi et al., 2020), viruses (van der Merwe et al., 2017; Filloux et al., 2018; Boykin et al., 2019; Della Bartola et al., 2020), and nematodes (Eccles et al., 2018; Knot et al., 2020).

Despite the great success, the MinION technique still presents some drawbacks. In fact, the portability of MinION comes at the expense of sequencing accuracy, as sequencing errors often range between 5 and 15% (Rang et al., 2018). Nevertheless, recent improvements in chemistry, as well as the use of consensus calling using bioinformatics tools, allowed to increase sequencing accuracy up to 97%, comparable with standard lab equipment (Vaser et al., 2017; van Dijk et al., 2018). A recently developed base caller, DeepNano-blitz (Boza et al., 2020), enables real-time data analysis without requirement of a powerful IT facility, increasing the possibility to deploy MinION sequencing in the field. However, the optimization, establishment, and standardization of methods for the quantitative evaluation of microbial composition in the environment are inevitable. PCR-based 16S rRNA analysis of bacterial community structure was shown to be subject to biases from the PCR-related conditions. These include the template concentration, DNA polymerase choice, number of cycles used, amplification reaction time, and the reaction temperature. When MinION sequencing was used after an accurate optimization of PCR condition, it was able to obtain bacterial community structures that were comparable in quality with MiSeq (Fujiyoshi et al., 2020).

Point-of-care sequencing is a still evolving technology that has shown a very fast development in portability, sequencing accuracy, and ease of operation. All these characteristics could make POC sequencing the gold standard of pathogen diagnosis in the next few years.

## Conclusion and Future Perspectives

The development of affordable and reliable methods for POC detection and identification of plant pathogens is not an easy task. There is an increasing demand for portable devices requiring minimal instrumentation that could perform a complete analysis directly in the field, even when used by personnel with only minimal training. This could bring great benefits to agriculture worldwide, as it would be possible to efficiently monitor wide cultivated areas for the presence of dangerous pathogens and detect them even before any symptom is actually visible. In the same way, it would be relatively easy to analyze imported plants and crops in order to prevent the spreading of pests and pathogens to new areas, where they could cause enormous damage to the local agriculture. There is also the need of pathogen monitoring in forestry and environmental applications, which often requires sampling in remote areas far away from laboratory facilities.

The nucleic acid-based methods are proving to be good candidates for POC pathogen detection, especially since new isothermal amplification protocols have been developed. These methods are not widely used for field screening or for the analysis of imported plant material, probably because technical difficulties still exist, especially when trying to develop a protocol that can be routinely used for the detection of different pathogens in different plant species. Also, the relatively high cost of some of the methods, especially when applied to large-scale analysis, can be a problem. Nevertheless, PCR-based and isothermal nucleic acid amplification protocols coupled with fast extraction methods and simple detection devices are being adopted for some pathogens and crops. The development of new portable and sensitive nucleic acid detection methods is still a growing field of research that, in the next years, will provide exciting possibilities to both researchers and farmers. In many cases, POC analysis can provide a fast disease detection, have minimal risk, low cost, and provide a high-quality diagnostic experience for the operator. However, traditional laboratory testing is generally more advanced and allows a more accurate analysis, validation, and recording of the results. There are clearly situations where each methodology excels. Therefore, the two methodologies are going to merge and integrate by the development of new technologies. The main challenges that need to be addressed for POC testing are the optimization of reliable methods for all the main pathogens threatening agriculture worldwide, the adaptation of the existing methods to the specific conditions that a given plant or pathogen requires, a robust comparison to traditional technologies, and encouraging the final users to replace the traditional detection methods with novel ones offering significant benefits.

## Author Contributions

Both authors conceived and prepared the manuscript, performed literature search, wrote and edited the manuscript. Both authors contributed equally to the article and approved the submitted version.

## Conflict of Interest

The authors declare that the research was conducted in the absence of any commercial or financial relationships that could be construed as a potential conflict of interest.

## Footnotes

1 https://ourworldindata.org/land-use

2 https://nanoporetech.com/products/minion
